# A Conserved Requirement for *Fbxo7* During Male Germ Cell Cytoplasmic Remodeling

**DOI:** 10.3389/fphys.2019.01278

**Published:** 2019-10-10

**Authors:** Claudia C. Rathje, Suzanne J. Randle, Sara Al Rawi, Benjamin M. Skinner, David E. Nelson, Antara Majumdar, Emma E. P. Johnson, Joanne Bacon, Myrto Vlazaki, Nabeel A. Affara, Peter J. Ellis, Heike Laman

**Affiliations:** ^1^School of Biosciences, University of Kent, Canterbury, United Kingdom; ^2^Department of Pathology, University of Cambridge, Cambridge, United Kingdom

**Keywords:** *Fbxo7*, PI31, proteasome, spermatogenesis, cell remodeling, germ cell

## Abstract

Fbxo7 is the substrate-recognition subunit of an SCF-type ubiquitin E3 ligase complex. It has physiologically important functions in regulating mitophagy, proteasome activity and the cell cycle in multiple cell types, like neurons, lymphocytes and erythrocytes. Here, we show that in addition to the previously known Parkinsonian and hematopoietic phenotypes, male mice with reduced Fbxo7 expression are sterile. In these males, despite successful meiosis, nuclear elongation and eviction of histones from chromatin, the developing spermatids are phagocytosed by Sertoli cells during late spermiogenesis, as the spermatids undergo cytoplasmic remodeling. Surprisingly, despite the loss of all germ cells, there was no evidence of the symplast formation and cell sloughing that is typically associated with spermatid death in other mouse sterility models, suggesting that novel cell death and/or cell disposal mechanisms may be engaged in Fbxo7 mutant males. Mutation of the *Drosophila* Fbxo7 ortholog, *nutcracker* (*ntc*) also leads to sterility with germ cell death during cytoplasmic remodeling, indicating that the requirement for Fbxo7 at this stage is conserved. The *ntc* phenotype was attributed to decreased levels of the proteasome regulator, DmPI31 and reduced proteasome activity. Consistent with the fly model, we observe a reduction in PI31 levels in mutant mice; however, there is no alteration in proteasome activity in whole mouse testes. Our results are consistent with findings that Fbxo7 regulates PI31 protein levels, and indicates that a defect at the late stages of spermiogenesis, possibly due to faulty spatial dynamics of proteasomes during cytoplasmic remodeling, may underlie the fertility phenotype in mice.

## Introduction

During spermiogenesis, round haploid spermatids undergo terminal differentiation to form spermatozoa, developing specialized organelles – the acrosome and flagellum – necessary for fertility and motility, respectively. This involves a dramatic morphological transformation, including nuclear compaction via the eviction of histones and their replacement with protamines, and elimination of the bulk cytoplasmic content of the developing cells. Until late spermiogenesis, spermatids remain connected by intercellular bridges, through which cytoplasmic constituents are shared among haploid spermatids (Braun et al., 1989; Ventela et al., 2003). Cytoplasmic shedding also removes these bridges and allows the individual sperm cells to separate in a process called individualization.

The disposal of excess cytoplasmic contents, including mitochondria and other organelles, is critical to many aspects of late spermatid differentiation (Sakai and Yamashina, 1989). In mammalian spermatogenesis, cytoplasmic processes from the supporting Sertoli cells invade the spermatid cytoplasm during late elongation to form the “mixed body.” Concurrently, deep invaginations known as “crypts” form within the Sertoli cell cytoplasm. Active transport of the spermatids to the base of the crypts enables the development of extensive cell–cell contacts between the Sertoli and germ cells. As remodeling progresses, branches of the invading processes engulf and phagocytose portions of the spermatid cytoplasm, resulting in the loss of around 50% of spermatid cytoplasmic volume prior to spermiation. Finally, at spermiation, the spermatids are ejected from the crypts and actively transported back to the tubule lumen. There, they shed their remaining cytoplasm as residual bodies, which are then also phagocytosed by the Sertoli cells (Kerr and de Kretser, 1974; Sakai and Yamashina, 1989; Russell et al., 1989). In mice, crypt entry initiates at spermatid step 14 (epithelial stage II), and the spermatids are most deeply invaginated at step 15 (epithelial stages IV–VI), before migrating back to the lumen during step 16 (epithelial stages VI–VIII). Processing of spermatid cytoplasm in preparation for phagocytosis by the Sertoli cells includes caspase activation (Blanco-Rodriguez and Martinez-Garcia, 1999; Arama et al., 2003; Cagan, 2003) and the degradation of cellular components by specialized variants of the proteasome (Zhong and Belote, 2007; Qian et al., 2013; Bose et al., 2014). The 20S catalytic core of a proteasome is a barrel-shaped assembly, comprised of α and β subunits. Three of the β subunits, β1, β2 and β5, have peptidase activity, while access of substrates into the core is controlled by α subunits, which recruit proteasome activators (PAs). The constitutively expressed proteasome consists of a regulatory 19S “lid” associated with a 20S core particle (Bochtler et al., 1999; Voges et al., 1999), but in particular cell types, including sperm, or under stress conditions, alternate proteasome configurations come into play (Kniepert and Groettrup, 2014).

*Drosophila* spermatogenesis differs from mammalian spermatogenesis in several ways. In particular, *Drosophila* germ cells develop in cysts containing synchronously developing germ cells, rather than in tubules with multiple generations of germ cells contacting a single Sertoli cell. Nevertheless, many aspects of spermiogenesis are conserved, including the involvement of proteasomes and caspases in cytoplasmic remodeling of spermatids into mature sperm. In *Drosophila*, each cell assembles an actin-based “individualization complex” at the base of the nucleus following nuclear elongation. These complexes then slide caudally along the flagella of a group of 64 interconnected spermatids, promoting their separation and the removal of most of their cytoplasm and organelles into a membrane-bound sack, the cystic bulge, eventually discarded as a waste bag – the equivalent of the mammalian residual body (Fabian and Brill, 2012). During individualization, proteasomes migrate ahead of the individualization complex.

In *Drosophila*, Nutcracker (*ntc*) protein is essential for spermatid individualization, and homozygous *ntc* null mutant flies are sterile. Spermatids in flies with a null mutation in *ntc* undergo apoptosis in late spermiogenesis at the point when individualization would normally occur. The spermatid apoptosis is associated with failure to form individualization complexes, failure to activate spermiogenesis-related caspases, and reduced proteasome activity. These defects have been ascribed to an interaction between Nutcracker and proteasome binding protein PI31 (Arama et al., 2007; Bader et al., 2010, 2011). PI31 was discovered as an *in vitro* inhibitor of proteasome activity (McCutchen-Maloney et al., 2000; Zaiss et al., 2002), which distinguished it from other proteasome regulators, like PA200, PA28, and 19S, which all activate the 20S proteasome. However, within intact cells, multiple studies on the effects of PI31 homologs on proteasome activity in different species including yeast, flies, plants and mammals suggest that PI31 regulation of the proteasome may be contextually and spatially regulated. Its effects range from subtle or no effect in some cell types to functioning as an inhibitor of immunoproteasome maturation, a proteasome activator and a proteasome transporter in others (Chu-Ping et al., 1992, 1994; Zaiss et al., 1999; McCutchen-Maloney et al., 2000; Zaiss et al., 2002; Kirk et al., 2008; Li et al., 2014; Shang et al., 2015; Yang et al., 2016; Merzetti et al., 2017; Corridoni et al., 2019; Liu et al., 2019). In flies, DmPI31 activation of the 26S proteasome is essential for sperm differentiation, and DmPI31 levels are greatly reduced in *ntc* mutant testes, indicating that DmPI31 requires a stabilizing interaction with *ntc* to achieve sufficiently high expression levels (Arama et al., 2007; Bader et al., 2010, 2011). However, while transgenic overexpression of DmPI31 in *ntc* mutant testes promoted caspase activation in germ cells, it did not restore the ability to form individualization complexes, and the flies remained sterile (Bader et al., 2011).

A mammalian ortholog of Nutcracker is Fbxo7, although there are likely to be functional differences between them given the inability of human Fbxo7 to rescue the sterility of *ntc* flies (Burchell et al., 2013). Fbxo7 is a multifunctional, F-box protein with distinct activities in different cell types. In human health Fbxo7 impacts on numerous pathologies, including Parkinson’s disease, cancer and anaemia (Laman, 2006; Soranzo et al., 2009; Di Fonzo et al., 2009; Ganesh et al., 2009; Paisan-Ruiz et al., 2010; Lomonosov et al., 2011; Ding et al., 2012; van der et al., 2012; Lohmann et al., 2015). At a molecular level, Fbxo7 functions as a receptor for SCF-type E3 ubiquitin ligases and also non-canonically, as a scaffolding chaperone for other regulatory proteins. Its effects are observable in NF-κB signaling, via cIAP and TRAF2 interactions (Kuiken et al., 2012), and in cell cycle regulation via Cdk6 activation and p27 stabilisation (Laman, 2006; Meziane et al., 2011; Randle et al., 2015; Patel et al., 2016). Fbxo7 has also been shown to interact with and ubiquitinate proteasome subunits, like PSMA2 (Bousquet-Dubouch et al., 2009; Fabre et al., 2015; Teixeira et al., 2016; Vingill et al., 2016). Because of its multi-functional nature affecting numerous cell types, several different mouse models of Fbxo7, including conditional and KO, have been engineered to study its effects in different cell types (Randle et al., 2015; Vingill et al., 2016; Patel et al., 2016; Stott et al., 2019; Joseph et al., 2019). The hypomorphic LacZ insertion mouse is anemic, shows thymic hypoplasia and T cell deficiencies, while conditional loss in different neuronal populations causes neurodegeneration. The KO mouse shows a pre-weaning lethality.

We report here that, like *ntc* flies, male mice with a reduced expression of Fbxo7 are infertile, and characterize the novel histology exhibited by this mutant. Given that the interactions of Fbxo7 and PI31 with each other and with the proteasome are known to be conserved between *Drosophila* and mammals (Kirk et al., 2008; Bader et al., 2011; Shang et al., 2015), we also investigated PI31 levels and localisation during testicular development. We show that Fbxo7 mutant mice have reduced PI31 levels in adult testes, but normal levels of constitutive proteasome activity in spermatids. This indicates that in mouse the fertility effects of Fbxo7 mutation are unlikely to be due to an overall deficiency in proteasome activity but may be due to a cell stage- or location-specific requirement for proteasome function.

## Results

### Fbxo7^LacZ/LacZ^ Mice Are Sterile Due to Azoospermia

**FIGURE 1:**
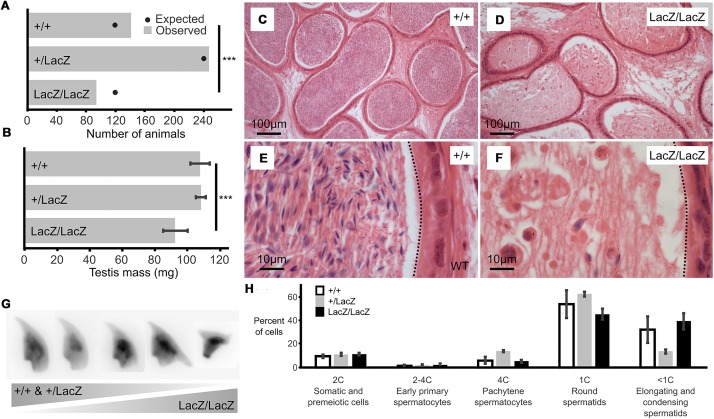
Male mice with reduced expression of Fbxo7 are sterile. **(A)** Colony data showing the genotypes of animals (*n* = 481) born from matings between heterozygous parents. All three potential genotypes are observed in the offspring, but the proportion of homozygous Fbxo7LacZ/LacZ offspring is lower than expected (^∗∗∗^ chi-squared goodness-of fit test *p* = 0.007 vs. Mendelian 1:2:1 expectation). **(B)** Average testis weight for each genotype (*n* = 6; WT and homozygous; *n* = 4, heterozygous LacZ; One-way ANOVA, ^∗∗∗^*p* = 0.001067). **(C)** Wild type cauda epididymis showing large numbers of stored sperm. **(D)** Fbxo7LacZ/LacZ cauda epididymis showing very few degenerating sperm. **(E,F)** High resolution zoom of sections **(C,D)**. Dotted line indicates the border of the tubule lumen in each view. **(G)** Montage of DAPI-stained sperm nuclei showing the spectrum of sperm morphologies present (see also Supplementary Figure S1). **(H)** FACS quantitation of testis cells according to DNA content as measured by propidium iodide staining (note that highly condensed spermatids and mature sperm were not quantitated); *n* = 4 WT, 2 heterozygous, 2 homozygous LacZ mice.

In the course of our investigations into the physiological functions of mammalian Fbxo7, we generated mice that are either heterozygous or homozygous for an allele of Fbxo7 containing a *LacZ* insertion between exons 3 and 4 of Fbxo7 (Randle et al., 2015; Patel et al., 2016). This insertion severely disrupts expression of all Fbxo7 isoforms but does not completely abolish it (Randle et al., 2015), and thus the phenotype(s) of the hetero- and homozygous animals can, respectively, be ascribed to moderate or severe under-expression of Fbxo7. In maintaining the colony of LacZ-transgenic animals, we observed that homozygous Fbxo7^LacZ/LacZ^ males never sired any offspring, while heterozygous males and all genotypes of female were able to produce litters. In heterozygous crosses, there was a mild deficit of homozygous offspring (Figure 1A), suggestive of a small degree of embryonic lethality in this genotype.

Initial characterization showed a significant reduction in mean testis weight for the Fbxo7^LacZ/LacZ^ compared to heterozygous and WT males (92.4 mg vs 108.2 and 107.7 mg; Figure 1B), indicative of abnormal testis development. Strikingly, there were virtually no mature sperm in the lumen of the epididymis of the Fbxo7^LacZ/LacZ^ males (Figures 1C–F), demonstrating that these males are sterile due to azoospermia. A very few residual sperm were retrieved from dissected epididymides of Fbxo7^LacZ/LacZ^ males, with a total yield of fewer than 1,000 cells per cauda epididymis, compared to a normal count of around 10^8^ sperm cells per cauda. The residual sperm were all grossly misshapen, and a high proportion of cells showed abnormal compression of the rear of the sperm head. Heterozygous Fbxo7^LacZ/+^ males also showed a slight increase in the frequency of abnormally shaped sperm, with the most severely deformed sperm resembling the homozygous phenotype (Figure 1G). Using a newly developed image analysis program for sperm morphometry (Skinner et al., 2019), we determined that the remaining sperm from Fbxo7^LacZ/LacZ^ males showed an average 14% reduction in cross-sectional area, with high morphological variability (Supplementary Figures S1, S2).

### Developing Fbxo7^LacZ/LacZ^ Spermatids Are Lost During Late Spermiogenesis

The relatively small magnitude of the change in testis weight suggested that any germ cell abnormality was likely to only affect later stages of germ cell development. To characterize the stage of germ cell loss in Fbxo7^LacZ/LacZ^ males, we carried out an initial assessment by FACS to see if there was any gross defect in meiotic progression (Figure 1H). There was no significant alteration in the ratio of cells containing 1C, 2C or 4C DNA content, respectively, representing haploid round spermatids, spermatogonia and primary spermatocytes, indicating no cell loss prior to the onset of spermatid elongation. Elongating and condensing spermatids/spermatozoa often appear as lower than 1C DNA content in FACS staining, but cannot be reliably quantified due to their high variability in staining parameters (Simard et al., 2015).

**FIGURE 2:**
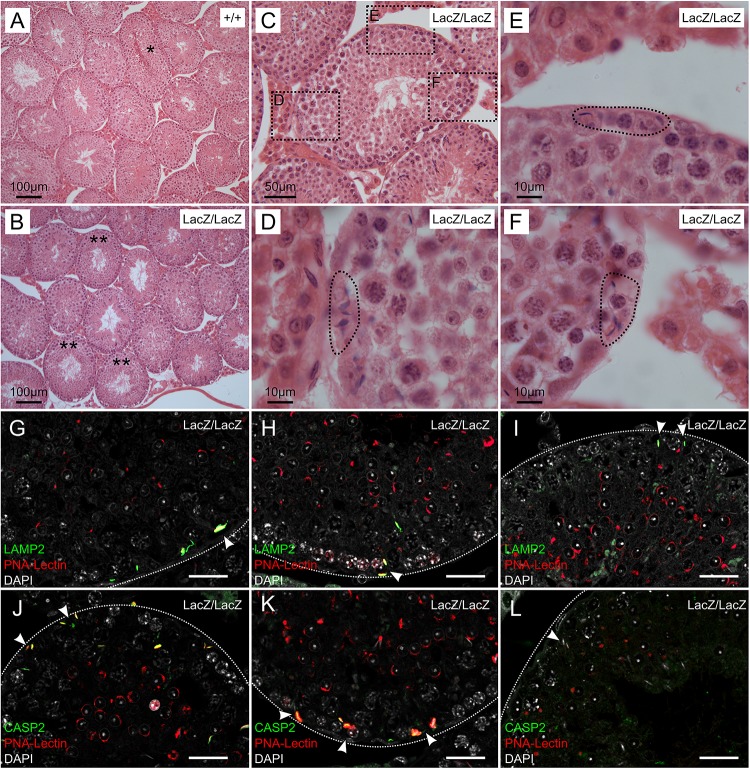
Immunochemical staining of caspase 2 and LAMP2 in Fbxo7LacZ/LacZ testis sections. **(A–B)** Low magnification view of H&E sections from wild type **(A)** and Fbxo7LacZ/LacZ testes **(B)**. ^∗^ In panel **(A)** indicates a tubule at stage VII-VIII with sperm heads lined up at the lumen awaiting release. These were never observed in Fbxo7LacZ/LacZ testes. ^∗∗^ In panel **(B)** indicates tubules with a layer of round spermatids but which lack elongating spermatids. **(C)** High magnification view of a Fbxo7LacZ/LacZ tubule lacking elongating spermatids: **(D–F)** Close up zoom from panel **C** at the indicated locations. The dotted outlines indicate “graveyards” containing multiple phagocytosed spermatid heads. **(G–I)** Immunofluorescent stains in Fbxo7LacZ/LacZ tubules for LAMP2 (green) with PNA-lectin (red) to stage the tubules and DAPI counterstain (gray). Phagocytosed cells marked by LAMP2 were visible at tubule stage VI as indicated by the extent of the lectin-stained acrosomal cap. **(J–L)** Immunofluorescent stains in Fbxo7LacZ/LacZ tubules for CASP2 (green) with PNA-lectin (red) to stage the tubules and DAPI counterstain (gray). Apoptotic cells marked by CASP2 were visible at tubule stage VI as indicated by the extent of the lectin-stained acrosomal cap. At tubule stage IV **(L)**, occasional mis-localised elongating spermatids were seen next to the basement membrane. These cells were not marked with CASP2 at this stage. For quantitation of spermatid mis-localisation, see Figure 3.

Histological examination of adult testes using hematoxylin and eosin staining (H&E) showed limited gross changes in testis structure (Figure 2 and Supplementary Figure S2). In both Fbxo7^LacZ/LacZ^ males and wild type (WT) males, pre-meiotic, meiotic and post-meiotic stages of germ cell development were all present in the testis parenchyma (Figures 2A,B). However, in Fbxo7^LacZ/LacZ^ testes, very few tubules showed sperm heads adjacent to the lumen (Figures 2B,C), suggesting that germ cells are lost prior to spermiation. Instead, testes from these males contained tubules with no (or virtually no) elongating cells, but where the first layer of spermatids was still round. These are tubules in the first half of the seminiferous cycle but where the late elongating cells have been lost. In these tubules, sperm heads were observed lying deep within the Sertoli cell cytoplasm near to the basement membrane, often in quite dramatic “graveyards” containing multiple cells in advanced stages of karyolysis (Figures 2D–F). Strikingly, however, we did not observe any formation of multinucleate symplasts or any sloughing of degenerating cells into the lumen (note also the lack of sloughed cells in the epididymis in Figure 1F).

### “Graveyards” of Phagocytosed Fbxo7^LacZ/LacZ^ Condensing Spermatids at Tubule Stage VI Are Positive for Caspase-2

Since the normal fate of arrested germ cells is apoptosis followed by either phagocytosis or cell sloughing, we used fluorescent immunohistochemical staining for caspase-2 and LAMP2 (Lysosome-associated membrane protein 2) to trace these processes. Caspase 2 is an apical caspase implicated in stress-mediated germ cell death (Zheng et al., 2006; Lysiak et al., 2007; Johnson et al., 2008), while LAMP2 labels late stage phagolysosomes. In this experiment, we also used fluorescently labelled peanut agglutinin (PNA) to label the acrosomes, allowing for more detailed tubule staging (Figures 2G–K). This showed that the cells in the “graveyards” were most prominent at tubule stage VI, and were positive for both LAMP2 and caspase-2. Lower-level caspase-2 staining was sometimes visible at this stage in spermatids located further from the basement membrane. We hypothesize these latter cells to be in the process of engulfment. The few remaining condensing spermatids near the lumen were still caspase-2 negative. Thus, the spermatids in the “graveyards” are apoptotic cells that have been phagocytosed by the Sertoli cells. We attempted to distinguish cells in later stages of apoptosis by immunohistochemical staining for caspase 3; however, this gave negative results in WT and mutant samples (data not shown). Activation of apical caspases independently of effector caspases is a known alternative cell death pathway in *Drosophila* germ cells, but has not yet to our knowledge been described in mammalian germ cells (Yacobi-Sharon et al., 2013).

In this experiment, we also noted occasional spermatids at earlier tubule stages (e.g., stage IV, Figure 2L) that appeared to be mis-localized, appearing next to the basement membrane, outside the peripheral ring of spermatogonia. Sperm heads are very rarely seen in this location in wild type testes unless they have been phagocytosed; however, these cells were generally negative for both caspase-2 and LAMP2, indicating that they were not yet apoptotic. Since LAMP2 only labels later stages of phagocytosis, we cannot exclude the possibility that these cells were in early stages of phagocytosis, and that phagocytosis in Fbxo7^LacZ/LacZ^ testes may precede the induction of apoptosis.

### Aberrant Localisation of Fbxo7^LacZ/LacZ^ Condensing Spermatids Initiates at the Onset of Cytoplasmic Remodeling, From Stages I-II Onward

**FIGURE 3:**
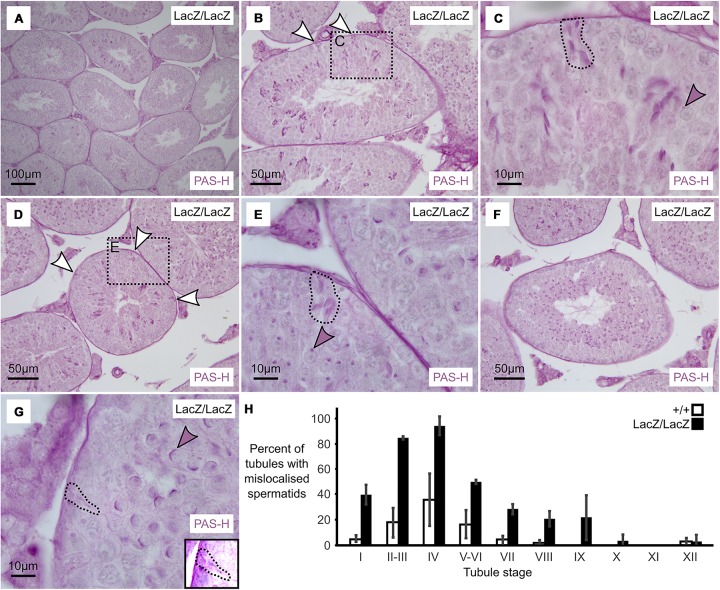
Massive loss of maturing sperm in Fbxo7 mutant males. **(A–G)** PAS-H staining of Fbxo7LacZ/LacZ testis sections used to quantitate spermatid mis-localisation. PAS marks the developing acrosome in purple, allowing both identification of the mis-localised elongating spermatids and also tubule staging. Panel **(A)** shows a low magnification view indicating general testis architecture. Panel **(B,D,F)** show complete tubules at stage II, IV, and VI, respectively, and white arrowheads indicate mislocalised elongating spermatids apposed to the basement membrane of the tubules – these are rarely visible at stage VI. Panel **(C,E,G)** show high magnification images at tubule stages II, IV and VI. Dotted outlines highlight mislocalised elongating spermatids, while shaded arrowheads indicate the developing acrosomes in the round spermatid layer, used to stage the tubules. Mislocalised spermatids were readily detected up to stage IV. After stage IV, the mislocalised cells were still present but began to lose their PAS staining were thus harder to detect. Panel **(G)** inset shows a rare example of a mislocalised cell remaining visible by PAS staining at stage VI. **(H)** Proportion of tubules containing at least one mis-localised cell at each tubule stage in wild type and Fbxo7LacZ/LacZ testes. Error bars indicate standard deviation across replicates (*n* = 3 animals per genotype).

We used periodic acid/Schiff/Hematoxylin (PAS-H) staging to quantify the onset of aberrant localisation of the condensing spermatids in the Fbxo7^LacZ/LacZ^ testes (Figures 3A–H). Here, the PAS staining labels the acrosome, allowing detection of spermatid location and staging. Only very light hematoxylin counterstaining was used to avoid obscuring the PAS signal. For maximal sensitivity in detecting the earliest stages of disorganization of the seminiferous epithelium, we scored any tubule with even a single spermatid observed at the basement membrane (i.e., appearing to be outside the Sertoli cell tight junctions) as positive.

We observed that mis-localization of late stage spermatids in the Fbxo7^LacZ/LacZ^ testes initiated as early as spermatid step 13–14 (tubule stage I–II), with the proportion of affected tubules apparently peaking at spermatid step 15 (tubule stage IV). From tubule stage VI onward, the phagocytosed spermatid heads were barely visible by PAS-H, most likely due to digestion of the epitopes detected by the PAS stain, and thus the apparent drop-off after stage IV is a technical artifact (note that dead cells at this stage remained visible via H&E and immunostaining; see Figures 1, 2). This contrasts with the immunostaining data where the mis-localized cells prior to stage VI were LAMP2 and CASP2-negative, but the phagocytosed cells at stages VI-VIII were strongly LAMP2 and CASP2 positive. The two experiments thus probe different aspects of the phenotype: mis-localization followed by apoptosis. Complete data from the PAS-H cell counting are supplied as Supplementary Table S2.

### PI31 Expression Is Reduced in Fbxo7^LacZ/LacZ^ Testes, but Proteasome Activity Is Unaltered

**FIGURE 4:**
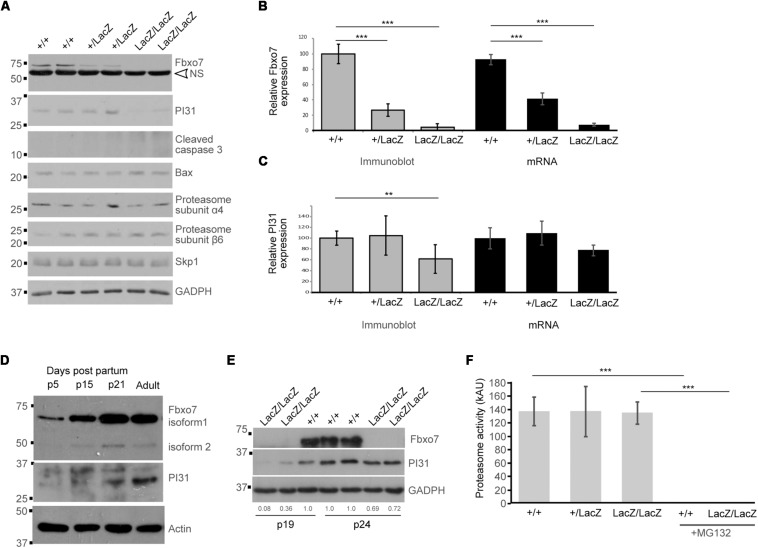
Decreased PI31 levels but normal proteasome activity in Fbxo7LacZ/LacZ testis. **(A)** Immunoblot analysis for various proteins in whole testes lysates from WT, heterozygous, and homozygous LacZ mice at 8.1 weeks of age, as indicated. NS = non-specific band in Fbxo7 (Santa Cruz Biotechnology, sc-271763) Western blot. **(B)** Quantitation of Fbxo7 expression from immunoblots, relative to GADPH loading control; gray bars; *n* = 10 WT, 8 heterozygous, 7 homozygous LacZ mice. RT-qPCR of Fbxo7 relative to three housekeeping genes (cyclophilin, GAPDH, and actin); black bars; *n* = 4 for each genotype). Images were analyzed in ImageJ. ^∗∗∗^*p* < 0.001 One-way ANOVA with Dunnett’s multiple comparisons test. **(C)** Quantitation of PI31 expression from immunoblots, relative to GADPH loading control; gray bars; *n* = 10 WT, 8 heterozygous, 7 homozygous LacZ mice. RT-qPCR of Psmf1/PI31 relative to three housekeeping genes (cyclophilin, GAPDH, and actin); black bars; *n* = 4 for each genotype). Images were analyzed in ImageJ. ^∗∗^*p* < 0.01 One-way ANOVA with Dunnett’s multiple comparisons test. **(D)** Immunoblot analysis for Fbxo7 (Laman Lab polyclonal antibody) and PI31 protein in whole testes lysates from mice harvested at the indicated days post-partum (p). **(E)** Immunoblot analysis for Fbxo7 (Santa Cruz Biotechnology, sc-271763) and PI31 protein in whole testes lysates from mice harvested at 19 and 24 days post-partum. Quantification of PI31 protein levels relative to WT GADPH levels for each sample is indicated. **(F)** Proteasome activity measured in whole testis extract for each genotype. Treatment with MG132 abolished the signal, confirming the specificity of the assay. *n* = 3 WT, 2 heterozygous, and 4 homozygous LacZ mice, each performed in triplicate ^∗∗∗^*p* < 0.0001.

The basis for sterility in *ntc*-mutant null flies is proposed to be the loss of a stabilizing interaction with DmPI31, leading to reduced proteasome activity (Bader et al., 2011). To address whether this relationship is conserved in spermatogenesis in mice, we tested the expression of Fbxo7 and PI31 in lysates made from testes of mature males (Figure 4A). As expected, the presence of the Fbxo7^LacZ^ allele caused dose-dependent decreases in Fbxo7 expression, as seen by both Western blot and qRT-PCR (Figures 4A,B). We note that for homozygous Fbxo7^LacZ/LacZ^ mice, expression of both mRNA and protein for Fbxo7 was reduced by approximately 94%. This is partial effect on Fbxo7 mRNA expression in testes is comparable to the that seen in liver (93%), cerebellum (80%), bone marrow (78%), and spleen (60%) (Randle et al., 2015).

PI31 protein levels were significantly reduced by 39% in adult Fbxo7^LacZ/Lac*Z*^ testes (Figure 4A), while a 23% reduction in mRNA levels was not statistically significant (Figure 4C). The fact that PI31 protein levels show a more pronounced decline than mRNA levels suggests that Fbxo7 has a role in stabilizing PI31 protein levels in adult testes. We therefore characterized the developmental profile of both proteins in normally developing wild type testes. Both Fbxo7 and PI31 were weakly detected at all ages by Western blot, indicating widespread low-level expression in the testis. Both, however, also showed strong upregulation between postnatal day 15 and day 21, concurrent with the first appearance of haploid spermatids in the testis (Figure 4D). PI31 was further upregulated between day 21 and adult testes, consistent with increased expression in later stage elongating/condensing spermatids. Finally, we examined Fbxo7 and PI31 levels in Fbxo7^LacZ/LacZ^ testes at days 19 and 24, i.e., at time points before the onset of germ cell mis-localization and loss. At 19 days of age, mutant testes showed a 64–92% reduction, while at 24 days, there was a ∼30% reduction in PI31 protein levels compared to wild type testes (Figure 4E). We conclude that Fbxo7 is required for PI31 stability in earlier stage germ cells.

To test whether the observed reduction in PI31 levels in adult testes led to decreases in proteasome activity, we conducted proteasome activity assays on whole testes from WT, heterozygous and homozygous *Fbxo7*^LacZ^ males. However, no reduction in proteasome activity was detected (Figure 4F). Consistent with these data, we observed no changes in the levels of core proteasome subunits α4 or β6 among the different adult WT and mutant testes by Western blot analysis (Figure 4A), indicating stable overall levels of proteasomes. Our attempts to measure PI31 levels and to conduct similar proteasome activity assays on elutriated cell populations were inconclusive due to the poor recovery of later stage spermatids from mutant testes (data not shown). These data indicated there were no major alterations in the overall levels of proteasome activity in testes from Fbxo7 mutant males.

Since Fbxo7 can form part of an E3 ubiquitin ligase, we also assayed for the levels of Skp1, the adaptor protein which recruits F-box proteins into SCF-type E3 ubiquitin ligases. Skp1 levels were unchanged, indicating that other SCF-type E3 ubiquitin ligases would be unaffected in mutant testes. Finally, we also examined levels of cleaved caspase 3 and the pro-apoptotic mediator Bax, both of which are implicated in germ cell death during the first wave of spermatogenesis (Russell et al., 2002; Said et al., 2004). We observed no changes in Bax levels between genotypes, and no expression of cleaved caspase 3, which was consistent with our inability to detect cleaved caspase 3 by IHC staining (data not shown). These data suggest that classical apoptotic pathways were not engaged during germ cell death in Fbxo7 mutant testes.

### Spermatoproteasome Localisation and Histone Removal From Spermatid Chromatin Are Unaltered in Fbxo7^LacZ/LacZ^ Testes

During spermatogenesis, in addition to the constitutive proteasome, alternative proteasomes are co-expressed. PA200-capped spermatoproteasomes promote the degradation of acetylated histones, which enables their removal from DNA and replacement with protamines, for enhanced nuclear compaction into the spermatid head (Gaucher et al., 2010; Qian et al., 2013). They contain an alternative α4-type proteasome subunit, α4s/PSMA8, a testis-specific subunit, which replaces its 20S counterpart, and enables the recruitment of an alternate lid, PA200. A second proteasome, known as the immunoproteasome, is also expressed and has alternate β-subunits, β1i, β2i and β5i, and a different regulatory 11S lid (Qian et al., 2013). As sperm differentiation requires major cellular remodeling and volume reduction, these alternate proteasomes are thought to play crucial roles in fashioning this specialized cell form (Zhong and Belote, 2007; Kniepert and Groettrup, 2014; Rathke et al., 2014).

**FIGURE 5:**
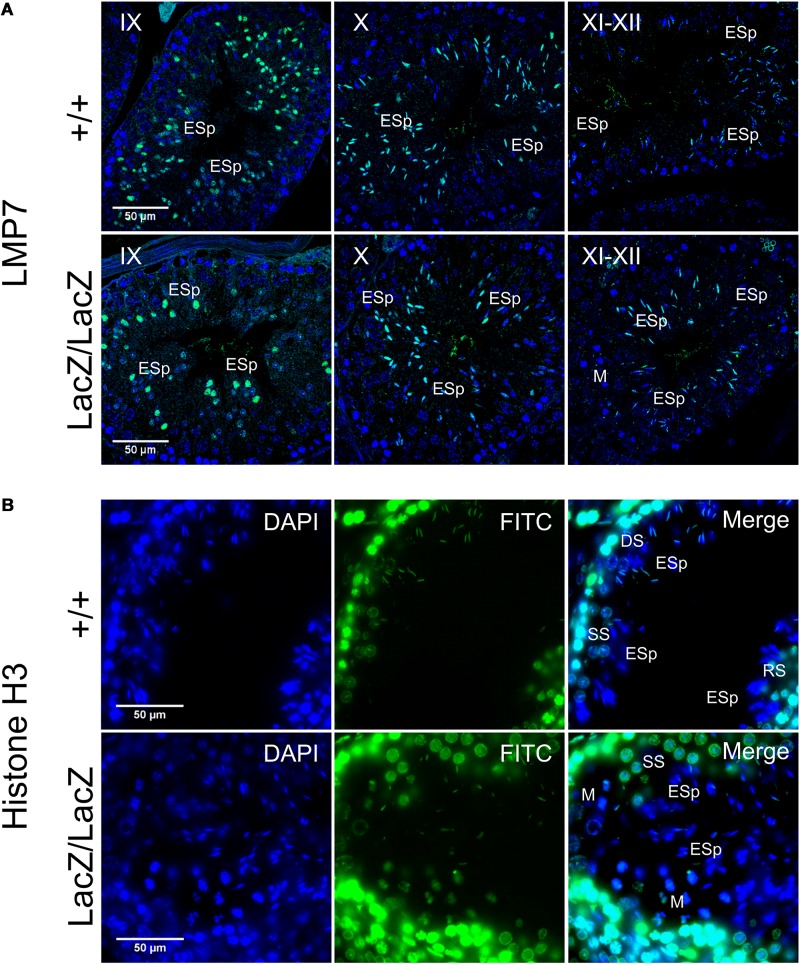
Immunochemical staining of LMP7 and histone H3 in Fbxo7LacZ/LacZ testis sections. **(A)** Immunochemical staining of LMP7 (FITC, green) in wild type and Fbxo7LacZ/LacZ testes with DAPI (blue) nuclear counterstain. Roman numerals indicate tubule stage. In both genotypes, nuclear LMP7 expression is first seen in early ES at mid-stage IX as the nuclei begin to elongate. This nuclear expression is highest at stage X, and then is lost during stage XI-XII as nuclei complete elongation. **(B)** Immunochemical staining of histone H3 (FITC, green) in wild type and Fbxo7LacZ/LacZ testes with DAPI (blue) nuclear counterstain. The wild type tubule shown is in transition between stages, with early stage XII (DS next to ESp) at upper left, mid stage XII (SS next to ESp) at lower left and stage XII/I border (M and RS next to ESp) at lower right. Nuclear H3 signal in ESp is still present at early stage XII, is restricted to the posterior of the nucleus in mid stage XII, and entirely lost by stage I. The Fbxo7LacZ/LacZ tubule shown is in mid stage XII, and the H3 signal in the ESp is absent or restricted to the posterior extremity of the nucleus, confirming the kinetics and spatial pattern of H3 removal are indistinguishable between genotypes. Key: DS = diplotene spermatocytes, SS = secondary spermatocytes, M = metaphase figures, RSp = round spermatids, ESp = elongating spermatids.

Although spermatoproteasome activity cannot be directly assayed independently of total proteasome activity, the spermatoproteasome has a key role in histone degradation during nuclear elongation (Kniepert and Groettrup, 2014). We therefore stained WT and mutant testes for LMP7 (β5i), a component of the immunoproteasome and spermatoproteasome which is not present in normal proteasomes, to determine whether this was altered by Fbxo7 deficiency (Figure 5A), and for histone H3 to determine whether the dynamics of histone removal was perturbed in the knockout males (Figure 5B). This showed no alteration in LMP7 localization in the mutant, with nuclear LMP7 signal being specific to stage IX-XII spermatids in both genotypes. There was also no delay in histone removal in the mutant, with all histone H3 signal being removed from the nucleus by the end of stage XII in both wild type and Fbxo7^LacZ/LacZ^ testes. Taken together with the overall proteasome assay data shown above, we conclude that both LMP7-containing proteasomes and normal proteasome activity are apparently unaffected by Fbxo7 deficiency in mouse testes.

**FIGURE 6:**
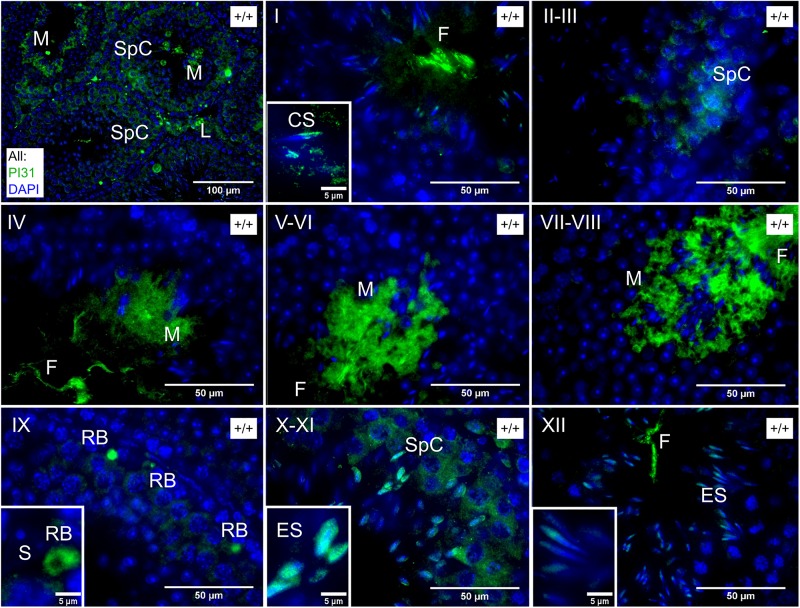
Immunochemical staining of PI31 localisation in wild type testes. Roman numerals indicate tubule stage. PI31 signal is cytoplasmic in Spc/M/RB/L but nuclear in ES and early CS. Key: SpC = spermatocyte, ES = elongating spermatid, CS = early condensing spermatid, M = mature or late condensing spermatid, RB = residual body, L = Leydig cell, S = Sertoli cell, F = flagellum of mature sperm.

Finally, while unfortunately, the available Fbxo7 antibodies do not work for immunohistochemical (IHC) staining in mouse testes, we investigated the spatial distribution of PI31 to determine whether this was consistent with a role in nuclear or cytoplasmic remodeling of spermatids. In wild type testes, PI31 was present in the cytoplasm of most cell types, becoming significantly stronger in the cytoplasm of late condensing spermatids from stage V onward and being retained into the residual bodies shed at stage VIII. In addition to the stage-specific cytoplasmic signal, PI31 also showed nuclear staining specifically in wild type elongating spermatids from stages IX through to XII (Figure 6). Thus, there is the potential for Fbxo7 and/or PI31 to regulate proteasomes during spermatid nuclear and/or cytoplasmic remodeling.

## Discussion

### The Mammalian Phenotype Associated With Fbxo7 Deficiency

Spermiogenesis is a multi-step process that transforms morphologically simple round spermatids into highly specialized mature sperm. It occurs in four successive phases, namely; (a) nuclear elongation and replacement of histones by transition proteins in tubule stages IX to XII, spermatid step 9–12), (b) migration of condensing spermatids into Sertoli cell crypts, protamination of sperm chromatin and cytoplasmic reduction by ∼50% in tubule stages I to VI, spermatid steps 13–15, (c) migration of the mature spermatids to the tubule lumen in tubule stage VII, spermatid early step 16, (d) spermiation, the release of fully formed sperm at tubule stage VIII, spermatid late step 16. In Fbxo7^Lac*Z*/Lac*Z*^ males, we observe a peculiar and very specific phenotype consisting of mass phagocytosis of condensing spermatids, occurring after nuclear elongation and prior to migration of spermatids back to the tubule lumen. This coincides with the cytoplasmic remodeling of the spermatids during steps 13–15, equivalent to the individualization stage of *Drosophila* sperm development. The requirement for Fbxo7 at this stage thus appears to be conserved from fruit flies to mammals.

The nature of the sterility phenotype in Fbxo7^Lac*Z*/Lac*Z*^ males is to our knowledge unprecedented in a mammalian system. Various null mutants with defects in cytoplasmic remodeling have previously been described, including Sept4, Spem1, Capza3, and Ube2j1 mutants. These all show either no phagocytosis or only limited phagocytosis of elongating spermatids during the first half of the cycle, followed by spermiation failure and spermatid retention at the lumen into stage IX and beyond (Kissel et al., 2005; Zheng et al., 2007; Geyer et al., 2009; Koenig et al., 2014). Further mutants have been described that show a step 13 block to spermatid development without defects in cytoplasmic remodeling, including the Bclw and Brd7 null mutants. In these mice, the arrested step 13 spermatids degenerate while still near the tubule lumen, followed by phagocytosis of large symplasts and other cell debris (Russell et al., 2001; Wang et al., 2016). In stark contrast to both of the above types of mutant, the Fbxo7^Lac*Z/LacZ*^ males showed complete phagocytosis of all developing spermatids with no detectable symplast formation, sloughing of degenerating cells into the lumen, or spermiation failure.

### How Are the Germ Cells Eliminated in Fbxo7^Lac*Z*/Lac*Z*^ Testes?

In Fbxo7^Lac*Z*/Lac*Z*^ testes, mis-localized condensing spermatids are visible by PAS-H staining at the basement membrane from tubule stage ∼I-II onward, and by stage IV almost 100% of tubules have mis-localized cells. At these early stages, however, mis-localized spermatids are negative for caspase-2 and LAMP-2, and retain their acrosomes (i.e., they are stainable by PAS), suggesting that they are not yet apoptotic and/or that phagocytic degradation has not yet initiated. By stage VI, however, the cells have lost their normal orientation, become positive for caspase 2 and LAMP-2, and karyolysis has initiated.

One possible scenario is that the early stages of mis-localization represent an abnormal deepening of the Sertoli cell crypts in mutant testes, and that the condensing spermatids have not yet been phagocytosed at this point. The trafficking of spermatids into and out of Sertoli cell crypts is governed by dynein-coupled motion of a specialized adherens junction complex between germ cell and Sertoli cell, known as the apical ectoplasmic specialisation (AES). If the AES is dissolved prematurely, the Sertoli cell may be unable to eject the spermatids from the crypts, leading ultimately to their death and phagocytosis.

An alternative scenario is that the phagocytosis initiates at stage I-II due to defects in germ cell remodeling, but that the engulfed cells remain alive and non-apoptotic for a short period after being phagocytosed. That is, the developing spermatids are “eaten alive” some time before finally dying. In this case, this would represent death by primary phagocytosis or “phagoptosis,” a relatively newly identified manner of cell elimination (Brown and Neher, 2012). Phagoptosis of elongating spermatids in Fbxo7^Lac*Z*/Lac*Z*^ testes would explain the absence of symplasts and the lack of sloughing of dead cells into the lumen, since in the case of phagoptosis, the eliminated cells would remain alive until they have already become trapped inside phagocytic vacuoles. Future studies to distinguish between these models using electron microscopy, in conjunction with immuno-labeling to resolve the intracellular machinery, are planned.

### Are the Consequences of Fbxo7 Deficiency Mediated by PI31/Proteasome Regulation?

In flies, *ntc* has been shown to be important for caspase activation and sperm individualization through enhancing DmPI31 stability, which acts as a proteasome activator. However, transgenic restoration of PI31 levels in *ntc* testes restored caspase activity but did not restore individualization complex assembly, suggesting that nutcracker and DmPI31 may both be necessary for sperm production (Bader et al., 2011). We and others have previously shown that the stabilising interaction between Fbxo7 and PI31 is conserved in many different cell types (Kirk et al., 2008; Bader et al., 2011; Shang et al., 2015; Merzetti et al., 2017). Consistent with this, we show here that PI31 protein levels are reduced in adult Fbxo7^LacZ/Lac*Z*^ testes. Importantly, PI31 protein levels are more strongly reduced than mRNA levels, indicating that this is a stabilizing effect of Fbxo7 on PI31 at the protein level.

The reduction in PI31 levels in Fbxo7^LacZ/LacZ^ testes is also seen in juvenile testes at days 19 and 24 days post-partum. At postnatal day 19, the most advanced germ cells are at the early round spermatid stage, while at day 24, the most advanced germ cells are early elongating (not condensing) spermatids. Thus, the reduction in PI31 precedes the appearance of condensing spermatids in the testis and is not simply a secondary consequence of the loss of late stage condensing spermatids in mutant testes.

In wild type testes, we show that PI31 and LMP7 are both present in the nucleus of step 10–12 spermatids, indicating that alternate proteasomes play a role in nuclear elongation. PI31 then shifts to the cytoplasm of step 13–16 spermatids at tubule stages I-VIII. The loss of germ cells in the knockout is thus coincident with this shift in PI31 localization from the nucleus to the cytoplasm. This is especially intriguing in the light of recent work showing that PI31 acts as an adaptor to facilitate proteasome transport in axons (Liu et al., 2019). One could imagine a role for PI31 in the re-localization of proteasomes in the remodeling cytoplasm. The spermiogenesis phenotypes could arise due to insufficient levels of PI31 causing deficiencies in localized requirements for proteasomes during remodeling but leaving overall proteasome activity intact.

Although we could not directly measure spermatoproteasome activity, whole-testis proteasome activity showed no changes in Fbxo7^LacZ/Lac*Z*^ testes. Other proteasome-related knockout males (PA200, PA28γ) have defects in multiple stages of germ cell development, both pre- and post-meiotic. The post-meiotic phenotypes of these knockout models include delayed histone replacement during nuclear remodeling and delayed spermiation, neither of which was seen in Fbxo7^LacZ/LacZ^ testes (Qian et al., 2013; Huang et al., 2016). Moreover, unlike Fbxo7^LacZ/Lac*Z*^ males, even the PA200/PA28γ double knockout males were able to produce substantial numbers of morphologically normal sperm in their epididymis (Huang et al., 2016). A knockout of the spermatoproteasome-specific subunit PSMA8 has recently been shown to lead to meiotic abnormalities and early spermatid arrest [Gómez Hernández et al., unpublished data, preprint^1^ ], unlike the late stage spermatid loss described in the present study.

Taken together, our data shows that the Fbxo7^LacZ/Lac*Z*^ male sterility phenotype differs from all other existing mouse knockouts related to proteasome function at both the histological and molecular levels. Hence, the Fbxo7 sterility phenotype appears not to be due to a generalized insufficiency of proteasome activity, although it is plausible that localized proteasome function in the cytoplasm is necessary during spermiogenesis, and our experiments have not addressed this.

### Potential Non-proteasomal Pathways Regulated by Fbxo7 That May Lead to Male Sterility

Fbxo7 is required for PINK1/Parkin-mediated mitophagy, a process that requires the fragmentation and engulfment of depolarized regions of the mitochondrial network (Burchell et al., 2013), and interestingly, both the *nutcracker* and *parkin* null flies show male sterility, with a specific defect during sperm individualization (Greene et al., 2003; Bader et al., 2010). In the *parkin* null mutant in *Drosophila*, a specialized mitochondrial aggregate present in insect sperm, known as the Nebenkern, failed to form, and spermatids failed to individualize suggesting that rearrangement of mitochondria is necessary for individualization. However, the mouse parkin null mutant is fertile with no known effects on germ cell development (Itier et al., 2003), and thus the sterility of Fbxo7 mutant males is unlikely to relate directly to its interaction with Parkin.

One possible explanation is that Fbxo7 is more generally involved with specialized cytoplasmic remodeling. In a similar vein to the sperm maturation defects, Fbxo7 has also been shown to be required during the final maturation steps of erythrocytes. We previously reported Fbxo7^LacZ/LacZ^ mice are anemic due to delayed mitophagy and defects in exiting cell cycle (Randle and Laman, 2016). Importantly, during this maturation step, macrophages in erythroblast islands phagocytose the shed organelles from maturing reticulocytes (Geminard et al., 2002; Zhang et al., 2015; Ovchynnikova et al., 2018), a process requiring the coupling of the autophagy and exocytosis pathways (Mankelow et al., 2015). Could this coupling be coordinated by Fbxo7? If so, then in a testis context, Fbxo7 may enable the fragmentation and isolation of portions of the spermatid cytoplasm to allow phagocytosis by Sertoli cells. In the absence of Fbxo7, failure to correctly package spermatid cytoplasm for elimination could instead lead to wrongful engulfment of complete cells and phagoptotic cell death.

As a third alternative but non-exclusive possibility, we note that the dead cells at tubule stage VI were strongly positive for caspase 2. TRAF2, a target of Fbxo7 ubiquitination (Kuiken et al., 2012), has recently been shown to bind to active caspase 2 dimers and ubiquitinate it to stabilize the activated complex (Robeson et al., 2018). Consequently, Fbxo7 deficiency could lead to over-activity of TRAF2 and subsequently signal the activation of caspase 2 precipitating germ cell death. Identifying the substrates of Fbxo7 underlying the unique phenotypes reported here is an area of future research.

## Conclusion

*Fbxo7* mutant mice exhibit a novel sterility phenotype unlike any previously described, in that total death and phagocytosis of all condensing spermatids occurs in the absence of typical hallmarks of spermatid apoptosis such as symplast formation and cell sloughing. The mis-localization of elongating spermatids initiates substantially before the appearance of markers of apoptosis and phagocytosis, indicating that aspects of spermatid trafficking into and out of Sertoli cell crypts may also be perturbed in these males. These males thus provide a new model of late spermiogenic failure, and an exciting new avenue to investigate cell remodeling, tissue remodeling and apoptosis in germ cell development.

## Materials and Methods

### Mice

Mice used in this study are *Fbxo7*^LacZ^ mice (*Fbxo7^tm 1*a(EUCOMM)Hmgu*^* on a C57BL/6J background) and experiments involving them were performed in accordance with the United Kingdom Animals (Scientific Procedures) Act 1986 and ARRIVE guidelines. Mice were housed in individually ventilated cages with unrestricted access to food and water, and 12-h day-night cycle. Animal licenses were approved by the Home Office and the University of Cambridge’s Animal Welfare and Ethical Review Body Standing Committee. Experiments were performed under the Home Office licenses PPL 80/2474 and 70/9001 (HL).

### Tissue Processing and Immunohistochemistry

Testes were fixed in Bouin’s fixative at 4°C overnight and embedded in paraffin. Sections were subjected to standard methods of hematoxylin/eos/in or periodic acid/Schiff/hematoxylin staining for histological examination. For immunohistochemical studies, sections were de-paraffinised in xylene and rehydrated through a graded ethanol series prior to blocking and antibody staining steps. Details of primary and secondary antibody concentrations are given in Supplementary Table S1. Antibody-stained sections were counterstained with DAPI and visualized via epifluorescence (PI31, Histone H3) or confocal fluorescence microscopy (Caspase 2, Lamp-2, LMP7). In some experiments, fluorescently conjugated peanut agglutinin from *Arachis hypogaea* (PNA-lectin) was included during the secondary antibody incubation step to visualize acrosomal morphology and facilitate staging of seminiferous tubules. Antibody validation and negative controls are shown in Supplementary Figure S3.

### Sperm Morphometric Analysis

Sperm were collected from two Fbxo7^LacZ/LacZ^, three Fbxo7^LacZ/+^, and two wild type males. The vasa deferentia and caudae epididymides were dissected from each animal, and the contents extracted into 1 mL PBS. Sperm were transferred to a microfuge tube, and tissue clumps were allowed to settle. Then sperm were transferred to a new tube and pelleted at 500 × *g* for 5 mins. The supernatant was removed, and the sperm fixed dropwise with 3:1 methanol-acetic acid. Sperm were again pelleted at 500 × *g* for 5 mins, and washed in fixative twice more. Samples were stored at −20°C. Fixed sperm nuclei were stained with DAPI and imaged using a 100× objective on a Nikon Microphot SA epifluorescence microscope equipped with a cooled CCD camera and appropriate filters. Images were captured using SmartCapture 2, exported in 8-bit tiff format and analyzed using the automated morphometric software Nuclear Morphology Analysis v1.13.7^2^ (Skinner et al., 2019). Hierarchical clustering was performed on nuclear shapes to group them into morphological categories, and the proportion of cells from each genotype in each category was calculated. The total numbers of nuclei analyzed for each genotype were 453 for Fbxo7LacZ/LacZ, 1225 for Fbxo7^LacZ/+^ and 756 for wild type.

### Flow Cytometry

Single-cell suspensions from whole testis tissue were pelleted by centrifugation, and resuspended in 1 mL of ice cold 80% ethanol/PBS while vortexing to disperse clumps. The suspended cells were fixed for at least 1 hr at 4^*o*^C. After fixation, cells were collected by centrifugation and washed once in PBS. The washed cell pellet was resuspended in 1 mL of a solution of 50 μg/mL propidium iodide (PI) staining and 10 μg/mL DNase-free RNase, and incubated for 10 min at 37°C, prior to analysis by flow cytometry (Beckman-Coulter, Inc.).

### Lysis and Immunoblotting

Whole testis tissue were lysed in RIPA buffer (50 mM Tris–HCl pH 7.6, 150 mM NaCl, 1% NP-40, 0.1% SDS, 0.1% Na deoxycholate, 1x protease inhibitors, 1 mM PMSF, 10 mM sodium fluoride, 1 mM sodium orthovanadate) (all from Sigma-Aldrich), and incubated on ice for 30 min with occasional vortexing. Cell debris was pelleted by centrifugation at 16,000 × *g* for 10 min at 4°C, and the supernatant retained. Protein concentration was determined via 96-well BCA assay (Pierce). Sample concentrations were standardized by dilution with lysis buffer. For Western blot, samples were mixed with equal volumes of 2x Laemmli buffer, denatured (95°C, 5 min), separated via SDS polyacrylamide gel electrophoresis (SDS-PAGE), and transferred onto polyvinylidenefluoride (PVDF) membrane (Millipore) using a semi-dry transfer system (Biorad). Membranes were blocked for 1 h with 5% non-fat, milk powder/PBS-Tween 20 (0.05%) (PBS-T), and then probed with primary antibody overnight at 4°C in 5% non-fat, milk powder/PBS-T. Membranes were washed in PBS-T and incubated with the appropriate HRP-conjugated secondary antibody in 5% non-fat, milk powder/PBS-T followed by further washes, and detection of HRP bound protein using enhanced chemiluminescence (ECL, GE Healthcare) and exposure onto X-ray film (Konica Minolta). Signal was quantified with background correction and normalized using ImageJ software (NIH, Maryland). Antibodies used in this study are provided in Supplementary Table S1.

### mRNA Isolation and RT-qPCR

Tissue was homogenised in 350 μL RLT buffer with β-mercaptoethanol and RNA isolated using RNeasy Plus kit (Qiagen) as per the manufacturer’s recommendations. One μg of mRNA was converted to cDNA using Quantitect reverse transcriptase (Qiagen), and then diluted 1:10 for subsequent RT-qPCR analysis using SYBR Green JumpStart Taq (Sigma) on a CFX Connect Real-Time PCR machine (Biorad). The following primers for *Fbxo7* (5′-CGCAGCCAAAGTGTACAAAG; 3′-AGGTTCAGTACTTGCCGTGTG) and *Psmf1*(PI31) (5′-CAATCATGCCACCTCTCTGA; 3′-CCGTCCTCATACTAG CAGGC) were used. RT-qPCR reactions were as follows: 95°C for 5 min then 45 cycles of 95°C for 30 s, 60°C for 30 s, 72°C for 30 s, followed by melt curve analysis to confirm a single PCR product was made. Relative gene expression was determined using relative standard curve method, data was normalized to three housekeeping genes (*Ppai, Gapdh, Actb*) as previously described [see (Birkenfeld et al., 2011; Meziane et al., 2011) for primer sequences], and expressed relative to WT levels.

### Counting of Mis-Localised Cells at Different Tubule Stages

Tubules were staged using periodic acid/Schiff staining to visualize the stages of acrosomal development (Oakberg, 1956; Russell et al., 1993). Every tubule in a complete testis cross-section was staged for three replicate males per genotype, by an observer blinded to the sample identity. Tubules were scored as positive if there were any mis-located elongating spermatid heads detected beyond the Sertoli cell tight junctions, within the outermost layer of nuclei in the tubule, and negative if there were no elongating spermatid heads within this layer. Tubules were also scored for the presence of “graveyards” defined as 2 or more mis-localized elongated spermatids enveloped by a single Sertoli cell. These definitions were chosen to maintain consistency across the seminiferous cycle, and their sensitivity is discussed in the main text.

### Measurement of Proteasome Activity

Assays for proteasome activity were performed using the Proteasome-Glo^TM^ Chymotrypsin-Like Cell-Based Assay Kit (Promega) according to the manufacturer’s protocol. Briefly, testes from 10 wk old mice were harvested and lysed in buffer (20 mM Hepes pH 7.6, 150 mM NaCl, 10% glycerol, 1% Triton X-100, 2 mM EDTA, 1 mM DTT) using a Dounce homogenizer in 10X the volume/weight of tissue. Protein concentration was measured, and lysates were diluted so that 100 μg of protein in 100 μL volume/well was loaded into a 96-well plate, and samples were plated in triplicate. Where applicable, samples were pre-incubated with MG-132 for 30 min prior to the addition of reagents, with protease inhibitors (Na_2_VO_4_, NaF, PSMF), or without inhibitors. Samples were equilibrated to RT for 15 min and then 100 μL of assay reagent was added. After a 10 min incubation, luminescence was measured in triplicate.

## Data Availability Statement

All datasets generated for this study are included in the manuscript/Supplementary Files.

## Ethics Statement

Mice used in this study are *Fbxo7*^LacZ^ mice (*Fbxo7^tm 1*a(EUCOMM)Hmgu*^* on a C57BL/6J background) and experiments involving them were performed in accordance with the United Kingdom Animals (Scientific Procedures) Act 1986 and ARRIVE guidelines. Animal licenses were approved by the Home Office and the University of Cambridge’s Animal Welfare and Ethical Review Body Standing Committee. Experiments were performed under the Home Office licenses PPL 80/2474 and PPL 70/9001 (HL).

## Author Contributions

HL and PE: conceptualization, methodology, and supervision. BS: software. CR, SA, SR, BS, DN, AM, EJ, JB, and MV: investigation. PE, HL, CR, SA, and BS: writing – original draft. BS, CR, PE, and HL: visualization. HL, PE, and NA: funding acquisition.

## Conflict of Interest

The authors declare that the research was conducted in the absence of any commercial or financial relationships that could be construed as a potential conflict of interest.

## Footnotes

1 https://www.biorxiv.org/content/early/2018/08/03/384354

2 https://bitbucket.org/bmskinner/nuclear_morphology/wiki/Home

## Supplementary Material

FIGURE S1 Sperm shape abnormalities are overrepresented in Fbxo7LacZ/LacZ animals compared to wild type and heterozygous animals. Sperm from all genotypes were clustered according to shape into three categories of normal, somewhat abnormal and severely deformed. Upper panel; representative DAPI-stained sperm nuclei from each cluster; middle panel; consensus shape of the cluster; lower panel; percent of each genotype within the cluster. The majority of the Fbxo7LacZ/LacZ sperm have severe abnormalities, compared with less than 10% of wild type or heterozygote sperm.

FIGURE S2 Sperm from Fbxo7LacZ/LacZ animals are mostly smaller than sperm from wild type and heterozygous animals, with higher variability (measured as the difference between each nucleus’ shape and the median shape).

FIGURE S3 **(A)** Immunoblotting using the antibody against human Fbxo7 (made in house) of duplicate samples of whole cell lysates made from SHSY5Y cells stably expressing with an empty vector or a short hairpin RNA targeting Fbxo7 expression. **(B)** Immunoblotting of samples of whole cell lysates made from U2OS cells stably expressing with an empty vector or three independent short hairpin RNAs targeting PI31 expression. **(C)** Representative examples of negative control immunohistochemical staining on wild type and homozygous LacZ testes, omitting the various primary antibodies, relating to images shown in Figures 2G–L, 5A,B. Arrows indicate mis-localised spermatid nuclei in the “graveyards.” **(D)** Representative examples of negative control immunohistochemical staining on wild type testes, omitting the PI31 primary antibody, relating to images shown in Figure 6. There was no specific germ cell staining, only residual auto-fluorescence in interstitial tissue between the seminiferous tubules, which is highlighted in these images due to the long exposure time. In particular, at stage X-XI the negative control showed no signal in elongating spermatid nuclei and minimal staining in spermatocyte cytoplasm (compare to Figure 6 lower middle panel). Similarly, at stage V-VI, there was no signal in the condensing spermatid cytoplasm (compare to Figure 6, central panel).

TABLE S1 Details of antibodies used in this study.

TABLE S2 All tubules were examined for a complete testis cross section for three males from each genotype. sgl, tubules with one or more singleton spermatid heads located at the basement membrane. gy, tubules with one or more “graveyards” consisting of 2 or more mislocalised spermatids lying within the same Sertoli cell.
