# Supplementary figures and images for: A Conserved Requirement for Fbxo7 During Male Germ Cell Cytoplasmic Remodeling
Source: Front Physiol. 2019 Oct 10;10:1278. doi: 10.3389/fphys.2019.01278 (PMC6795710; doi:10.3389/fphys.2019.01278)

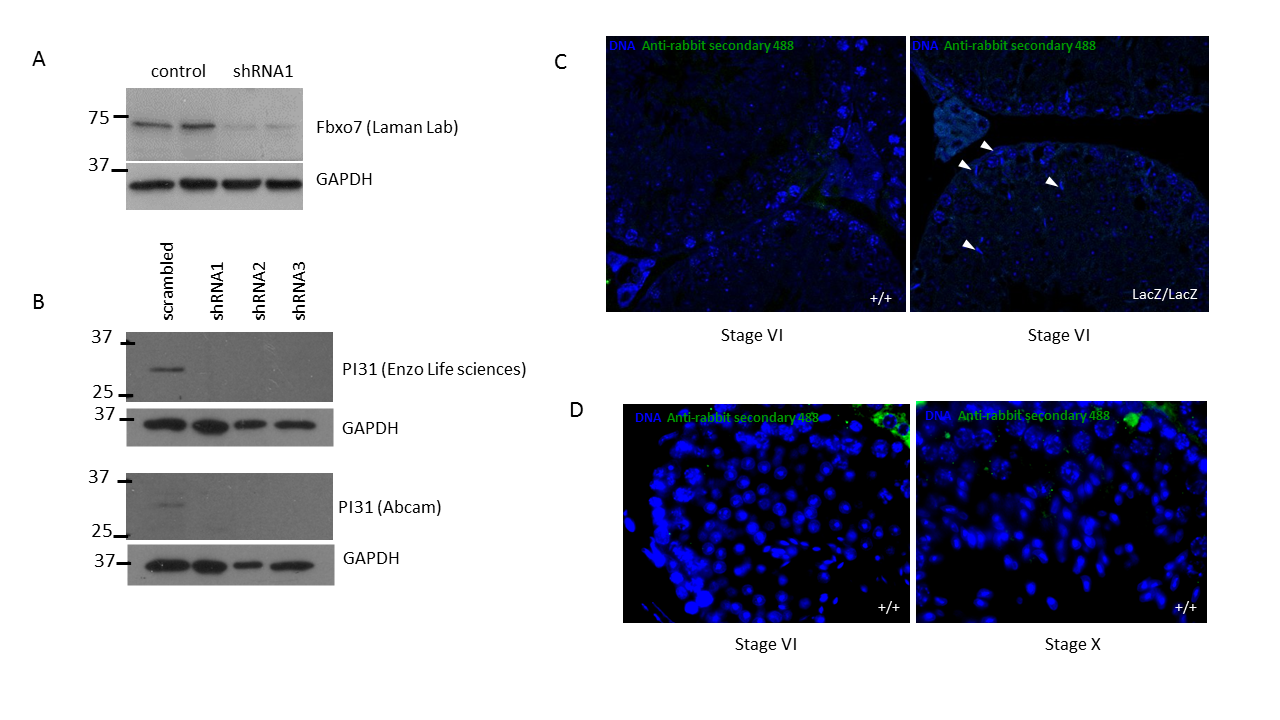

Supplement: FIGURE S3 — (A) Immunoblotting using the antibody against human Fbxo7 (made in house) of duplicate samples of whole cell lysates made from SHSY5Y cells stably expressing with an empty vector or a short hairpin RNA targeting Fbxo7 expression. (B) Immunoblotting of samples of whole cell lysates made from U2OS cells stably expressing with an empty vector or three independent short hairpin RNAs targeting PI31 expression. (C) Representative examples of negative control immunohistochemical staining on wild type and homozygous LacZ testes, omitting the various primary antibodies, relating to images shown in Figures 2G–L, 5A,B. Arrows indicate mis-localised spermatid nuclei in the “graveyards.” (D) Representative examples of negative control immunohistochemical staining on wild type testes, omitting the PI31 primary antibody, relating to images shown in Figure 6. There was no specific germ cell staining, only residual auto-fluorescence in interstitial tissue between the seminiferous tubules, which is highlighted in these images due to the long exposure time. In particular, at stage X-XI the negative control showed no signal in elongating spermatid nuclei and minimal staining in spermatocyte cytoplasm (compare to Figure 6 lower middle panel). Similarly, at stage V-VI, there was no signal in the condensing spermatid cytoplasm (compare to Figure 6, central panel). [file Image_3.TIF]
